# The Blinin Accumulation Promoted by CbMYB32 Involved in *Conyza blinii* Resistance to Nocturnal Low Temperature

**DOI:** 10.3390/ijms24087143

**Published:** 2023-04-12

**Authors:** Ming Yang, Min Zhou, Mengdan Shu, Zhengqi Han, Ruiqi Ma, Yuting Chen, Tianrun Zheng, Hui Chen

**Affiliations:** 1 College of Life Science, Sichuan Agricultural University, Ya’an 625014, China; 2 Traditional Chinese Medicine Planting Institute, Chongqing Academy of Chinese Materia Medica, Chongqing College of Traditional Chinese Medicine, Chongqing 402760, China

**Keywords:** *Conyza blinii*, unique terpenoid blinin, transcriptional regulation, virus-induced gene silencing, nocturnal low temperature resistance

## Abstract

Blinin, a unique terpenoid from *Conyza blinii* (*C. blinii*), benefits our health even though this is not its primary function. Physiological and ecological studies have found that the great secondary metabolites participate in important biological processes and relate to species evolution, environmental adaptation, and so on. Moreover, our previous studies have shown that the metabolism and accumulation of blinin has a close correspondence with nocturnal low temperature (NLT). To find out the transcriptional regulation linker in the crosstalk between blinin and NLT, RNA-seq, comparative analysis, and co-expression network were performed. The results indicated that CbMYB32 is located in a nucleus without independent transcriptional activation activity and is probably involved in the metabolism of blinin. Furthermore, we compared the silence and overexpression of CbMYB32 with wild *C. blinii*. Compared with the overexpression and the wildtype, the CbMYB32 silence line lost more than half of the blinin and detected more peroxide under NLT. Finally, as a characteristic secret of *C. blinii*, it is reasonable to infer that blinin participates in the NLT adaptation mechanism and has contributed to the systematic evolution of *C. blinii.*

## 1. Introduction

Exposure of plants to prolonged cold temperatures can alter the fluidity of the cell membranes, leading to irreversible damage to the cells [1]. Low temperatures inhibit the growth and development of plant chloroplasts, reducing the intensity and activity of photosynthesis, decreasing stomatal conductance and intercellular CO_2_ concentration, and affecting net photosynthetic efficiency and downstream photosynthetic production [2,3,4]. Low temperature affects plant reactive oxygen metabolism, which induces an increase in hydroxyl radicals, hydrogen peroxide (H_2_O_2_), and malondialdehyde (MDA) content, producing varying degrees of oxidative stress [5,6].

Plant secondary metabolites are closely linked to environmental stresses. Taking anthocyanin as an example, anthocyanin in the plant epidermis absorbs part of the light energy and provides some photoprotection to the leaves [7]. The stability of photosynthesis helps maintain the balance of plant cellular sugar stores, ensuring cellular osmotic pressure and the conversion of intercellular metabolites [8]. Overexpression of *AtMYB75* and *AtMYB12* results in the overaccumulation of flavonoids and attenuates the accumulation of reactive oxygen species in vivo under oxidative and drought stresses [9]. Overexpression of *PAP1* in *fls1* knockout mutants leads to a dramatic increase in anthocyanin, which enhances osmotic stress tolerance in Arabidopsis [10].

Medicinal plants usually grow slowly and have low content of active ingredients, leading to shortage of medicinal resources [11]. By simulating the natural environment, plants can be induced to synthesize more secondary metabolites. Low temperature enhances the accumulation of podophyllotoxin of *Dysosma versipellis* (*D. versipellis*) by upregulating podophyllotoxin pathway genes [12]. The content of flavonoids increases after low temperature treatment in *Tetrastigma hemsleyanum* (*T. hemsleyanum*) [13]. In addition, after a low temperature stimulating, the accumulation of ginsenosides can be enhanced in *Panax ginseng* adventitious roots [14]. Different chilling treatments stimulate the accumulation of different types of ginsenosides, which is necessary for the process of ginsenoside biosynthesis against low temperature [15]. AaMYC2 was found to be a positive regulator of artemisinin biosynthesis of *Artemisia annua* (*A. annua.*) [16]; the expression of 2-oxoglutarate-dependent dioxygenase (2-ODD) corresponds to tanshinone biosynthesis [17], which will contribute to increase secondary metabolite production through transgenic methods.

As a biennial endangered herb, *Conyza blinii* (*C. blinii*) is distributed in a high-altitude valley with large temperature difference and often suffers from nocturnal low-temperature stress (NLT). Blinin is a unique diterpene of *C. blinii* [18] which has proved as an indicator response to the environment in our previous experiments [19,20]. We found that after simulating NLT, the accumulation of terpenoids increased, which could enhance the quality of medicinal materials and alleviate the resource shortage of *C. blinii* [21]. In this experiment, RNA-seq was used to analyze the dynamic changes of terpenoid metabolism-related genes, further exploring the potential regulatory factors and the reasons for the increase in blinin in NLT.

## 2. Results

### 2.1. RNA Sequencing Reveals the Key Process Response to NLT

**Figure 1:**
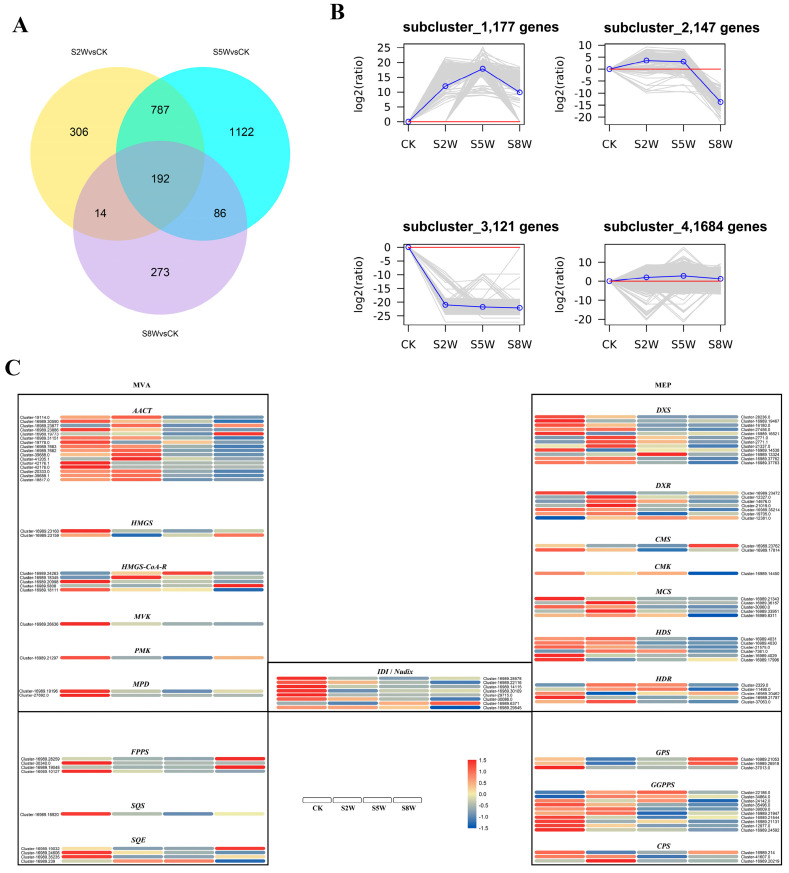
*Conyza blinii* transcriptome under NLT. The number of differentially expressed genes in S2W, S5W, and S8W. The intersecting part represents the common genes (**A**). All clusters were divided into four subclusters according to the expression trend. Above the red line is the up-regulated gene, and below the red line is the down-regulated gene (**B**). Gene expression of key enzymes in MEP and MVA metabolism pathways. Red represents high expression and blue represents low expression (**C**).

A total of 177 genes were up-regulated and 121 genes were down-regulated after RNA sequencing of *C. blinii* treated with NLT (Figure 1B). There were 192 common differentially expressed genes (DEGs) in S2W (stress 2 week), S5W (stress 5 week), and S8W (stress 8 week) (Figure 1A). After GO enrichment of the differentially expressed genes in S2W and S5W, all DEGs were annotated into three parts: biological process (BP), molecular function (MF), and cellular component (Cc). However, a total of 33 genes have been annotated as programmed cell death, and 47 genes have been annotated as photosynthetic genes (Figure S1D). Furthermore, all the DEGs were also clustered by KEGG pathway analysis. The most significant enrichments of the S2W and S5W upregulated DEGs were in ‘plant hormone signal transduction’, ‘phenylpropanoid biosynthesis’, and ‘plant-pathogen interaction’. In addition, only ‘plant-pathogen interaction’ was significant enrichened in S8W (Figure S1A–C). Blinin and saponin were synthesized by two metabolic pathways, MEP (methylerythritol phosphate, MEP) and MVA (mevalonic acid, MVA), which were isolated by organelles. The expression of *Nudix* hydrolase gene, which controls the exchange of MEP and MVA substances, showed a gradual downward trend. RNA sequencing showed that the key enzyme genes of the terpenoid metabolism were downregulated during the whole NLT. Gene expression in CK and S2W was higher than in S5W and S8W (Figure 1C).

### 2.2. Gene-Terpenoid Association Analysis

In this analysis, 14585 genes were divided into 26 gene expression modules according to gene expression. Then the 26 gene expression modules were associated with the number of adaxial/abaxial GTs, saponin accumulation, and blinin accumulation in leaves of *C. blinii.* The grey modules had extremely significant positive correlation with GTs and saponin accumulation. The orange modules had extremely significant positive correlation with blinin accumulation. The purple and grey modules had extremely significant positive correlation with the number of adaxial and abaxial surfaces GTs, respectively. Moreover, the purple modules had extremely significant positive correlation with the GT number. The cyan and pink modules showed extremely significant negative correlation with the GT number (Figure S2B).

### 2.3. The Co-Expression Network of TFs and Gene-Terpenoid

**Figure 2:**
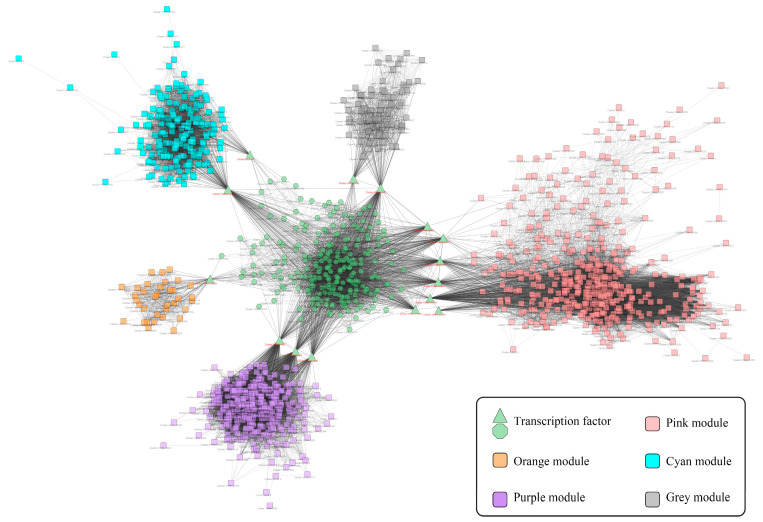
Gene-terpenoid network of *C. blinii* in NLT. Co-expression modules of terpenoid metabolism genes clustered. The different colors represent different gene modules. Green modules represent transcription factors, and other modules represent genes regulated by transcription factors. Potentially related genes are connected by gray lines.

A total of 214 differentially expressed transcription factors were detected in NLT. Among them, there were 20 unique transcription factors in the S2W stage, 82 unique in the S5W stage, 18 unique in the S8W stage, and 94 overlapping transcription factors (Figure S2A). Combined with the TF network and the gene-terpenoid association analysis, the TFs co-expression network of *C. blinii* in NLT was established (Figure 2). It should be noted that *cluster-16989.32228* and *cluster-16989.6391* are hub-linker TF linking orange module and grey module, respectively. This suggests that *cluster-16989.32228* and *cluster-16989.6391* may be related to terpenoid metabolism in NLT.

### 2.4. Screening of Transcription Factors Involved in Terpenoid Metabolism under NLT

**Figure 3:**
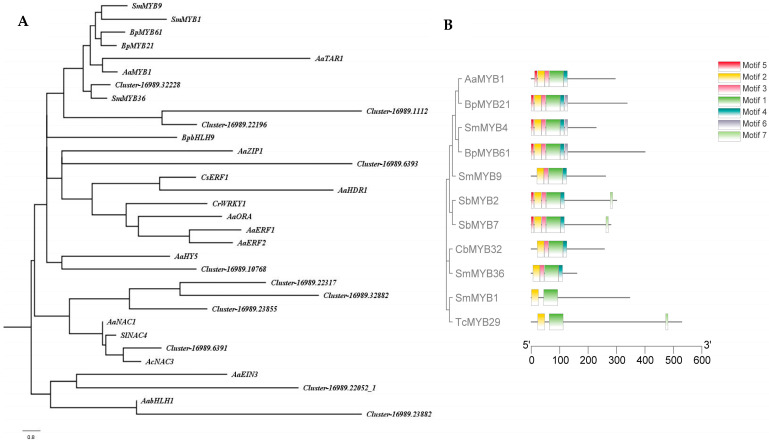
Phylogenetic analysis. Phylogenetic tree with TFs of *C. blinii* and other TFs have been reported involved in terpenoid metabolism within *Artemis iaannua*, *Salvia miltiorrhiza*, *Scutellaria baicalensis*, etc (**A**). Motif and phylogenetic tree with *CbMYB32* and other MYB TFs. Different colors show different motifs (**B**).

The hub-linker TFs and the reported genes regulating terpenoid metabolites were analysed for phylogenetic tree. *Cluster-16989.32228* and *SmMYB36* which regulates the synthesis of tanshinone were clustered into one branch. Furthermore, it was found that *cluster-16989.32228* has the same motif sequence as *SmMYB36*. Therefore, *cluster-16989.3228* was identified as a MYB transcription factor named *CbMYB32* (Figure 3).

### 2.5. The Expression Pattern Analysis of CbMYB32

**Figure 4:**
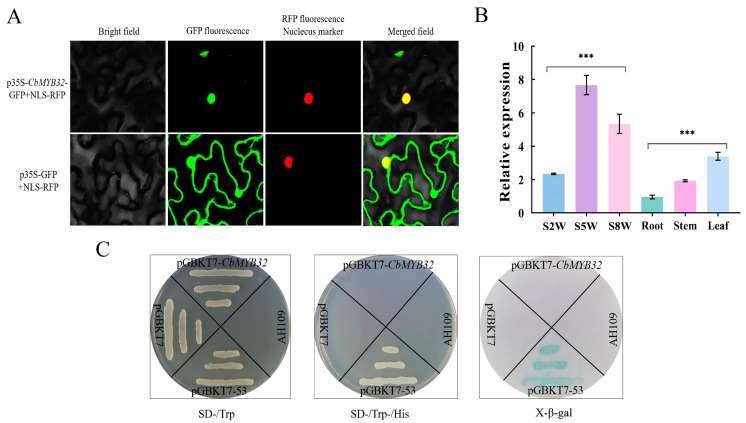
Expression pattern analysis of CbMYB32. Colocalization of p35S-*CbMYB32*-GFP in tobacco epidermal cells (**A**). The relative expression of *CbMYB32* in NLT and different plant tissues (**B**). Three independent biological repeats were set for each group. Asterisk indicates significant differences between each group (*** *p* < 0.001). Transcriptional activation assays of full length *CbMYB32* fused with the GAL4 DNA-binding domain (GAL4DB) in yeast (**C**). (-/Trp) indicates selective medium lacking Trp, (-Trp/-His/) indicates selective medium lacking Trp, His. Yeast growing on SD-Trp/-His/ soiled medium dyed by X-β-gal.

RT-qPCR was used to analyze the expression difference in *CbMYB32* plant tissues and NLT. It was found that the expression of *CbMYB32* was the highest in leaf tissue and reached the maximum value in this experiment at S5W (Figure 4B). By constructing a *CbMYB32*-eGFP vector, the green fluorescence signal was successfully located in the tobacco mesophyll nucleus (Figure 4A). In addition, yeasts containing *CbMYB32*-pGBKT7 couldn’t grow on the SD-Trp/-His solid medium plate, indicating that CbMYB32 didn’t have independent transcriptional activation activity (Figure 4C).

### 2.6. CbMYB32 Regulates Blinin Metabolism under NLT

**Figure 5:**
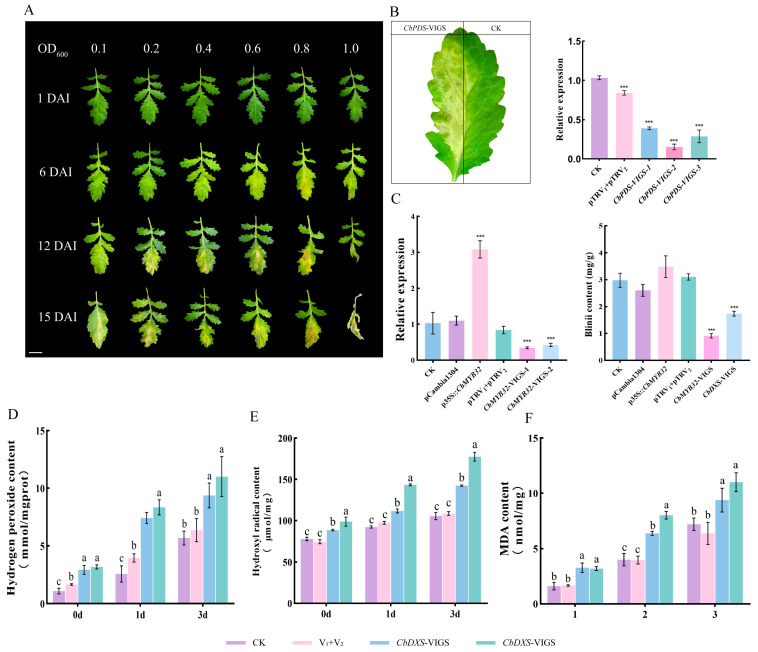
Agrobacterium-mediated transient transformation of *C. blinii*. Development of *CbPDS* transgenic leaves. DAI: days after infected. Leaves will bleach when the *CbPDS* is silenced. Bar = 1 cm. (**A**). *CbPDS*-VIGS leaf phenotype. Three samples were randomly selected to detect changes in expression after *CbPDS* was silenced. The pTRV_1_ + pTRV_2_ group indicating the empty vector control (**B**). The relative expression of *CbMYB32* treated with OE (overexpression) and VIGS under NLT for 3 days. Changes of blinin content after OE and VIGS under NLT for 3 days. The pCambia1300 indicating the empty vector control (**C**). Determination of hydrogen peroxide content under NLT (**D**). Determination of hydroxyl radical content under NLT (**E**). Determination of Malondialdehyde (MAD) content under NLT (**F**). Three independent biological repeats were set for each group. Asterisk indicates significant differences between CK and experimental groups at the same time (*** *p* < 0.05). Different letters indicate significant differences at the *p* < 0.05 level when comparing different experimental groups according to a one-way ANOVA.

Here, we have tried to establish the *C. blinii* VIGS method. Silencing of the reporter gene *CbPDS* accelerated leaf bleaching, but high concentrations of Agrobacterium made the leaves appear necrotic (Figure 5A). *CbPDS* gene expression was significantly suppressed after 15 days when the OD_600_ of the resuspension was 0.1 (Figure 5B).

Agrobacterium-mediated transient transformation of *C. blinii* leaves was used to verify whether CbMYB32 has regulatory effect on blinin. The infected *C. blinii* experienced 3 days of NLT induction; the expression of CbMYB32 significantly increased in the OE group (p35S::CbMYB32) and significantly decreased in the *CbMYB32*-VIGS group. It was difficult for the *CbMYB32*-VIGS group to accumulate blinin under NLT (the same results were also seen in the *CbDXS*-VIGS group); in contrast, the blinin content increased in the OE group, indicating that CbMYB32 has a regulatory effect on blinin metabolism under NLT (Figure 5C).

### 2.7. Blinin Maintains Stable Reactive Oxygen Metabolism under NLT

In addition, we found that when the *CbMYB32* and *CbDXS* genes were silenced, the synthesis of blinin in *C. blinii* was inhibited under NLT. In this situation, the contribution of blinin to the NLT tolerance of *C. blinii* is more likely to be explored. The results showed that the activities of OH· (Figure 5D), H_2_O_2_ (Figure 5E), and MDA (Figure 5F) all increased more in the VIGS group (*CbMYB32*-VIGS and *CbDXS*-VIGS group) than in the control group, which indicates that blinin could maintain the balance of reactive oxygen species metabolism in *C. blinii* under NLT.

### 2.8. Effect of SA on the Genes of Terpenoids Metabolic Pathway Enzymes

KEGG enrichment showed that ‘plant hormone signal transmission’ was detected in S2W-S5W. Based on our previous experiments, the regulatory relationship between the SA signal and the terpenoid metabolic was verified by exogenous hormone experiments [19]. We found that SA could upregulate the expression of *CbDXR* in the MEP pathway, and all the MEP pathway genes in this experiment were induced by SA + FDT. In addition, we also found that *CbMYB32* was induced by SA and inhibited by FDT. Thus, we speculated that the SA signal may be the regulatory signal of the MEP metabolic pathway (Figure S3).

## 3. Discussion

In previous experiments, it was found that NLT could increase the proportion of blinin in terpenes. At the early stage of NLT, *C. blinii* maintain relatively stable photosynthesis through fluctuating the expression of photosynthesis genes, which provides sufficient substrate and energy for the secondary metabolism. However, for the whole NLT process, the expression of the enzyme genes of the terpenoid metabolism showed a gradually decreasing trend. Terpenoids not only lack an effective degradation pathway, they can only be transported to the extracellular through transport proteins [22,23,24]. Excessive accumulation of secondary metabolites in plant cells could trigger programmed cell death [25]. In addition, IPP and DMAP are dephosphorylated and phosphorylated by Nudix, affecting MVA and MEP metabolism, which is the key to regulating the balance of the terpenoid metabolism. In our RNA sequencing results (Figure 1), the Nudix hydrolase genes have been already kept in a low activity at S2W, isolating the MEP and MVA metabolic pathways from each other prematurely, which will generate feedback regulation leading by the accumulation of downstream metabolites. These may be the reason why MEP and MVA metabolism showed a low activity in the later stage of NLT.

The results of RNA sequencing confirmed our judgment on the physiological process of *C. blinii* under NLT. Within 0-S3W, *C. blinii* might be in the preparation stage of sensing and capturing external signals. Within S4W-S6W, *C. blinii* might be in stage of response and adjustment to the external environment (most DEGs in this stage). Within S7W-S9W, *C. blinii* formed the primary tolerance and relatively stable metabolic activity, which is called the stable stage.

The gene-terpenoid network found that *CbMYB32* was the hub-linker TF linking the orange module. The phylogenetic tree showed that *SmMYB36* was the closest gene to *CbMYB32*. SmMYB36 has been demonstrated to interact with enzyme gene promoters or other transcription factors, directly or indirectly regulating the synthesis of tanshinones but inhibiting the synthesis of phenolic acids [26,27]. The phenomenon was discovered that a transcription factor interacting with several pathway genes was also found in other medicinal plants [28]. CbMYB32 functions as a promoter of blinin accumulation was demonstrated by our experiments, but its mechanism needs further verifying. Cold stress response–related MYB TFs have been reported in different species [29,30,31]. CaMYB306 represses the transcriptional activity of the calcineurin class B-interacting protein kinase gene 13 (CaCIPK13), affecting reactive oxygen species (ROS) system, which negatively regulates cold tolerance in pepper. Overexpression of the MYB-like gene *VaAQ* could improve cold tolerance through promoting the accumulation of osmoprotectants in grapevine (*Vitis vinifera* L.) [32]. The disruption of blinin synthesis alters the balance of reactive oxygen metabolism in *C. blinii* under NLT. Plant secondary metabolites are versatile and can act as effective regulators of plant growth and defense [33].

Our previous experiments inferred that ABA and SA signals might be highly correlated with oleanolic acid and blinin [19]. SA has a positive effect on resisting external low temperature [34]. SA mediates the expression of plant immune genes under low temperature to keep plant adaptability [35]. Salicylic acid can increase the metabolic activity of the terpene trilactones (TTLs) of *Ginkgo biloba* [36] and can interact with jasmonic acid to induce the flavonol glycoside accumulation of *G. biloba* cells [37]. Therefore, SA directly or indirectly participates in various biological pathways to maintain plant adaptability under low temperature.

**Figure 6:**
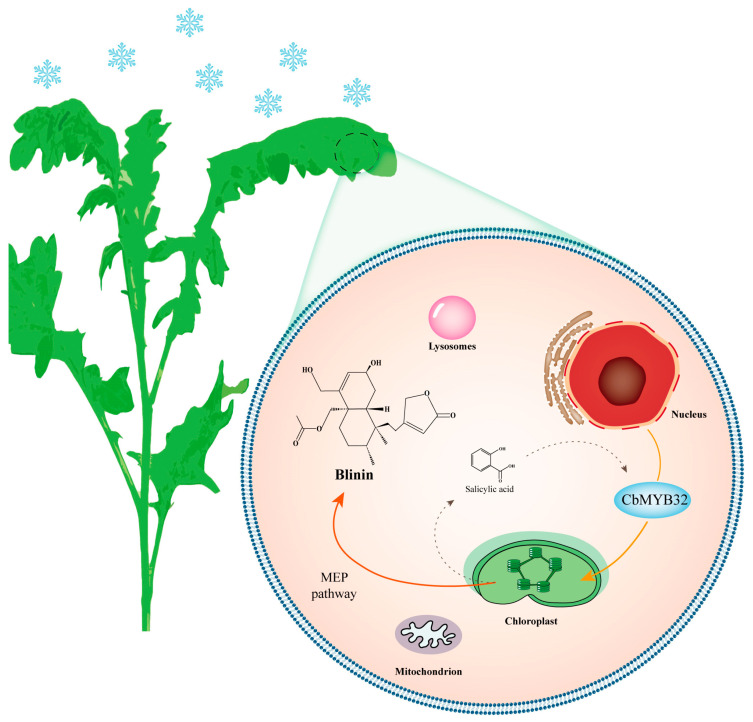
A model for NLS in *C. blinii* mediated by CbMYB32. Under NLS, CbMYB32 can enhance the terpenoid metabolism activity of *C. blinii* by activating blinin biosynthesis in the MEP pathway, which may connect with the SA signal pathway. The solid line represents the upstream stress signal transduction. The dashed line indicates that there may be an activation reaction.

In this study, we revealed the terpenoid metabolism changes of *C. blinii* during NLT through RNA sequencing and established the gene-terpenoid network. CbMYB32 was screened and found to be able to positively regulate the accumulation of blinin, which may be related to SA signal transduction (Figure 6). Clearly, the details of more gene-terpenoid response pathways need to be further explored. Our experiments also provide a basis for improving the medicinal quality of *C. blinii* through genetic means.

## 4. Materials and Methods

### 4.1. Plant Cultivation and NLT Treatment

Plant cultivation management and the NLT stress methods of *C. blinii* were mentioned in our previous study [19]. Plant materials were placed at 4 °C from 17:00 to 9:00 for 9 weeks and collected at 9:00 every other week. Two-month-old seedlings were selected for transient transformation. The relative humidity of the laboratory was 50–70%. All of the plants were managed under a long-day photoperiod (16 h: 8 h, light: dark).

### 4.2. RNA-Seq and Different Expression Gene Analysis

The RNA was extracted from the samples using TRNzol Universal Total RNA Extraction Reagent (Tiangen, Beijing, China). Eukaryotic mRNA was enriched with oligo (DT) magnetic beads to obtain the final cDNA library. Illumina HiSeq sequencing was performed. The library concentration was determined using qPCR to ensure that the effective library concentration was greater than 2 nM. Leave sequencing samples were set with 3 biological repeats, and then Illumina HiSeq sequencing was performed.

After sequencing the raw data, we filtered the raw data for low quality sequences to obtain clean reads. The transcriptome reference sequences of *C. blinii* were spliced by Trinity [38] for subsequent gene prediction, functional annotation, and expression analysis. Because there is no reference sequence of the whole genome of *C. blinii* at present, the gene function annotation of the transcriptome sequence was compared and annotated using NCBI (https://www.ncbi.nlm.nih.gov/, accessed on 12 November 2022), Pfam (http://pfam.sanger.ac.uk/, accessed on 12 November 2022), KOG/COG (http://www.ncbi.nlm.nih.gov/COG/, accessed on 12 November 2022), and Swissprot (http://www.ebi.ac.uk/uniprot/, accessed on 12 November 2022).

The transcriptome sequence (Genbank ID: SUB6240213, SUB9380850) has been uploaded to NCBI. Differential expression analysis was performed for NLT using the DEseq R package. Genes with adjusted *p* values < 0.05 were designated differentially expressed. Then GOseq package and KOBAS software were used to analyse the GO function enrichment and KEGG enrichment of the differential genes.

### 4.3. Differential Transcription Factor Screening and Co-Expression Network Analysis

Based on the different expression transcription factors (TFs), the correlation of differentially expressed transcription factors was calculated using the Hmisc and reshape2 of the R. On the basis of gene expression, all the DEGs were clustered into different modules through the WGCNA program. Using the terpenoids metabolism data in our previous studies [19,20] as a reference, the accumulation of adaxial surface GTs, abaxial surface GTs, saponin, and blinin was used to calculate the correlation between gene modules. We combined these with the co-expression network and the correlation of genes to terpenoids, building a hub-linker TFs network.

### 4.4. Phylogenetic Tree

The reported TF sequences regulating terpenoids were retrieved from NCBI (Table S2). Combined with the transcriptome gene of *C. blinii*, MEGA7 software was used to conduct the maximum quasi-natural method phylogenetic analysis. Motif analysis: The output. xmL file was downloaded to the MEME website: (https://meme-suite.org/meme/, accessed on 3 December 2022), and TBtools software was used for visual analysis.

### 4.5. Gene Cloning and Vector Construction

The full-length sequence of *CbMYB32* was amplified by primer (Table S1) and con- structed in pCambia1300 and pGBKT7 vector, respectively, by homologous recombination method (Vazyme, Nanjing, China, C115). Clone strain selection DH5α (WeiDi, Shanghai, China). PCR amplification conditions: 98 °C, 10 s; 61 °C,10 s; 72 °C, 30 s.

### 4.6. Subcellular Localization of the CbMYB32

The *CbMYB32* full-length CDS without the stop codon was amplified with *CbMYB32*-eGFP primer pair (Table S1) and inserted into the pCambia1300-eGFP vector to constitute CbMYB32-eGFP fusion expression vector. The fusion vector was introduced into DH5α and transiently expressed in leaves of *N. benthamiana* (1-month-old) by the infiltration method. The infected tobacco was cultured for 24 h in the dark and then transferred to a light incubator (25 °C, 16 h: 8 h, light: dark). After 48 h, the tobacco leaves were photographed by a confocal laser-scanning microscope (Olympus).

### 4.7. Transcriptional Activation of the CbMYB32 from C. blinii

The full-length CDS of *CbMYB32* was inserted into a pGBKT7 vector containing the DNA-binding region of GAL4. The vector plasmid was transferred to the AH109 strain and cultured according to the manufacturer’s instructions (WeiDi, Shanghai). The yeast on the selective medium lacking Trp (-Trp) solid medium plate was activated onto the medium lacking Trp and His (-Trp/-His/) solid medium plate. Dying with X-β-gal reagent after yeast grows for 3–5 days. If the yeast grew and turned blue, it was a sign of activation activity.

### 4.8. Agrobacterium Tumefaciens-Mediated Transient Transformation of C. blinii Leaves

Transient overexpression: *CbMYB32*-pCambia1300 vector was transferred to GV3101 Agrobacterium (WeiDi, Shanghai). YEB liquid medium was used to culture the Agrobacterium. After removal of supernatant, the resuspension buffer (100 mL buffer contains 1 M MES-KOH, 1 M MgCl_2_, 0.1 M AS acetosyringone) was added to the bacteria precipitation until the OD_600_ to 0.3–0.5. We injected the mixture from the abaxial surface of *C. blinii* leaves with 1 mL syringe.

VIGS of *C. blinii*: The two reporter genes are *CbPDS* (phytoene desaturase gene) and *CbDXS* (1-deoxy-D-xylose 5-phosphate synthase), which are both from the transcriptome database of *C. blinii*. The SGN-VIGS website (https://vigs.solgenomics.net/, accessed on 10 December 2022) was used to design a 200 bp VIGS specific silencing fragment, and this fragment was inserted into a pTRV_2_ vector. Afterwards the pTRV_2_ and pTRV_1_ were transferred to Agrobacterium GV3101.YEB liquid medium was used to culture the bacteria. After removal of supernatant, the resuspension buffer (100 mL buffer contains 1 M MES-KOH, 1 M MgCl_2_, 0.1 M AS acetosyringone) was added to the bacterial precipitation until OD_600_ to 0.1. After mixing pTRV_2_ and pTRV_1_ in 1:1 volume, we injected the abaxial surface with syringe *C. blinii* leaves.

The infected *C. blinii* were cultured for 24 h in the dark and then transferred to a light incubator (25 °C, 16 h: 8 h, light: dark) for 7–15 days.

### 4.9. Physiological Indices Measurements

The contents of OH·, H_2_O_2_, and MDA were evaluated using assay kits (Nanjing Jiancheng Bioengineering Institute, Nanjing, China). The HPLC detection method of blinin was mentioned in our previous study [19,39].

### 4.10. Plant Hormone Treatment and RT-qPCR

We sprayed exogenous hormones on 3-month-old *C. blinii*. The concentration of hormones and inhibitors referred to our previous experiments [20]. In this experiment, the concentrations of SA and ABA were 100 μM. The concentrations of ABT and FDT were 10 μM. The samples were collected after 24 h and 48 h. The real-time quantitative PCR methods were mentioned in our previous study [40].

## 5. Conclusions

In conclusion, the CbMYB32 transcription factor was involved in the accumulation of blinin *within C. blinii* in NLT, which may contribute to a faster adaptation of *C. blinii* to NLT stress.

## Supplementary Materials

The following supporting information can be downloaded at: https://www.mdpi.com/article/10.3390/ijms24087143/s1.
