# Supplementary material for: The Blinin Accumulation Promoted by CbMYB32 Involved in Conyza blinii Resistance to Nocturnal Low Temperature
Source: Int J Mol Sci. 2023 Apr 12;24(8):7143. doi: 10.3390/ijms24087143 (PMC10139108; doi:10.3390/ijms24087143)
Supplement: Supplementary file 1 [file ijms-24-07143-s001.zip › ijms-2329371-supplementary.pdf]

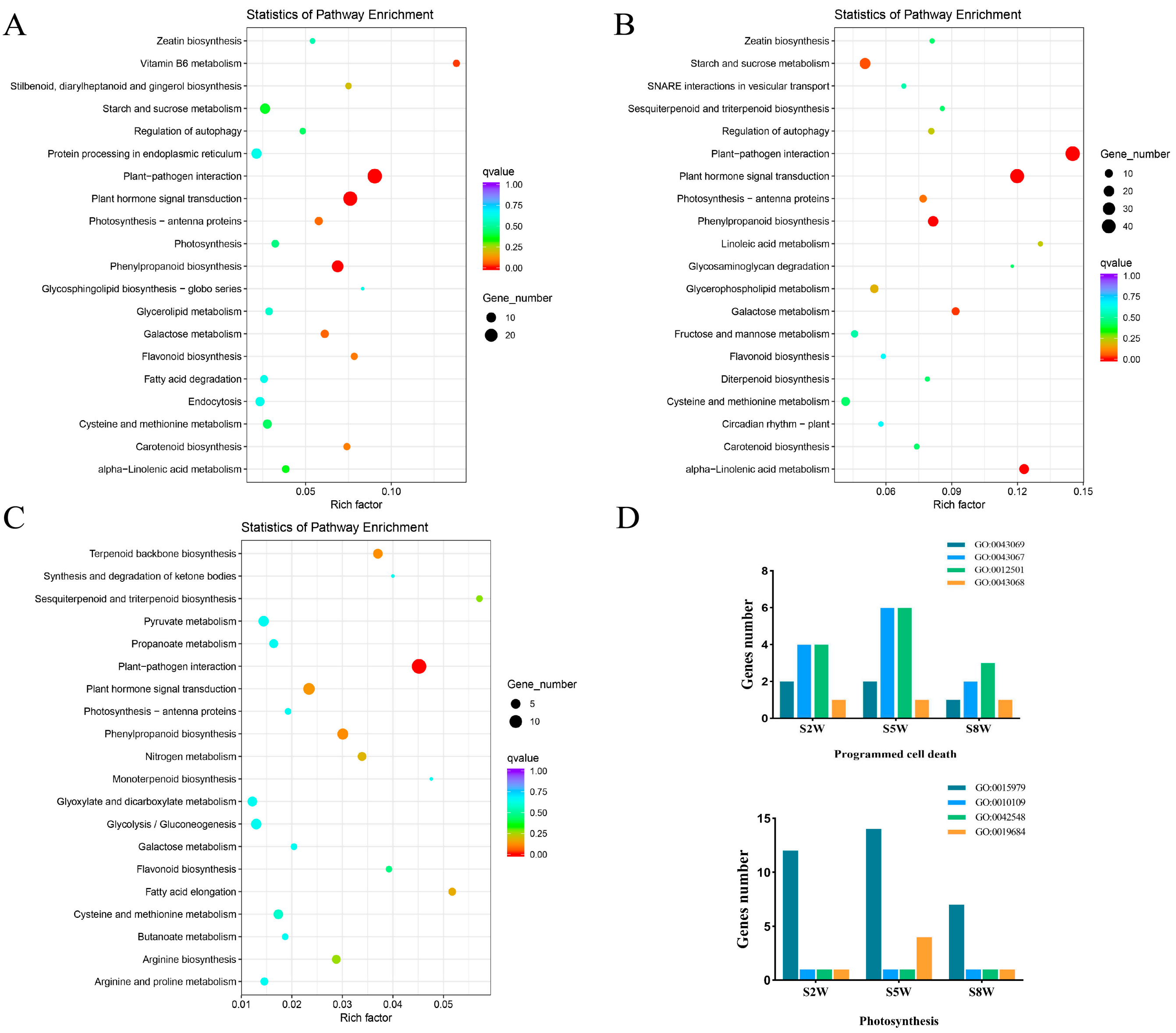

Supplemental Figure S1. KEGG enrichment analysis of up-regulated genes in S2W (**A**), S5W (**B**), S8W (**C**). The y-axis indicates the KEGG annotation information; the x-axis indicates the enrichment factor. Genes involved in programmed cell death (PCD) and photosynthesis annotated by GO (**D**).

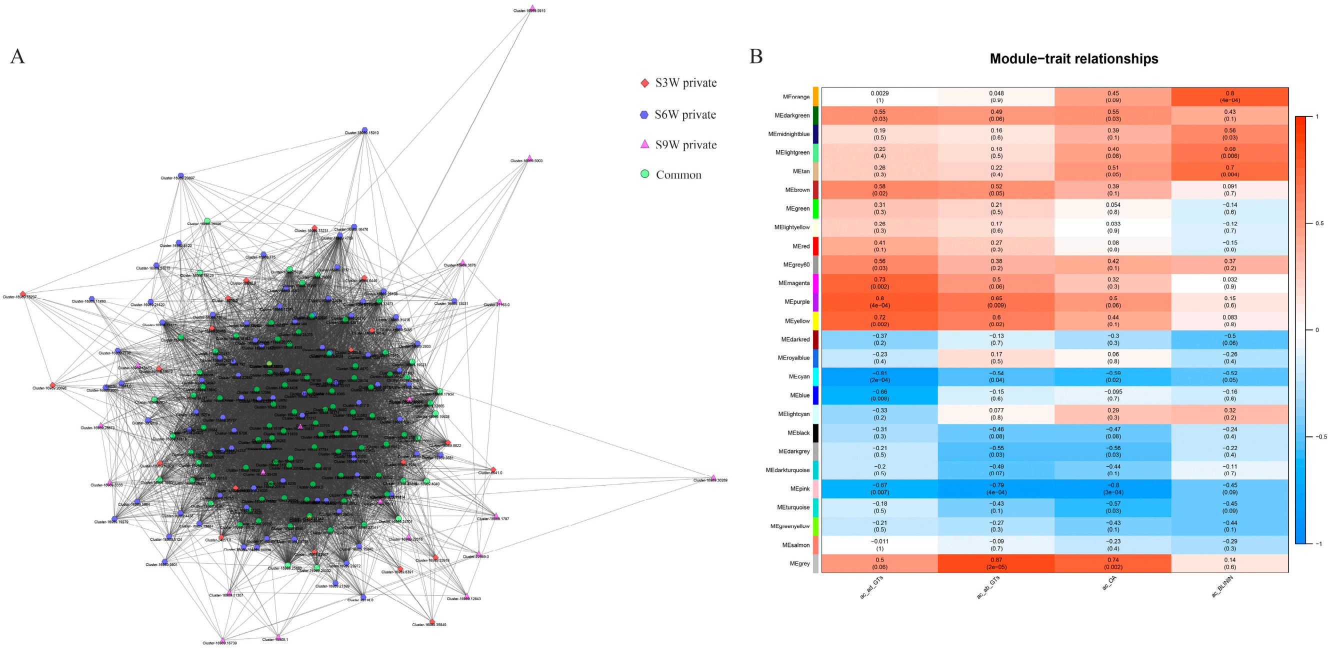

Supplemental Figure S2. Gene-terpenoid network. Co-Expression network of differential transcription factors for NLS in *C. blinii.* (**A**). Correlation analysis between epigenetic characteristics and gene modules (**B**). **ac_ad_GTs**: The accumulation of adaxial surface GTs; **ac_ab_GTs**: The accumulation of abaxial surface GTs; **ac_saponin**: The accumulation of saponin; **ac_blinin**: The accumulation of blinin.

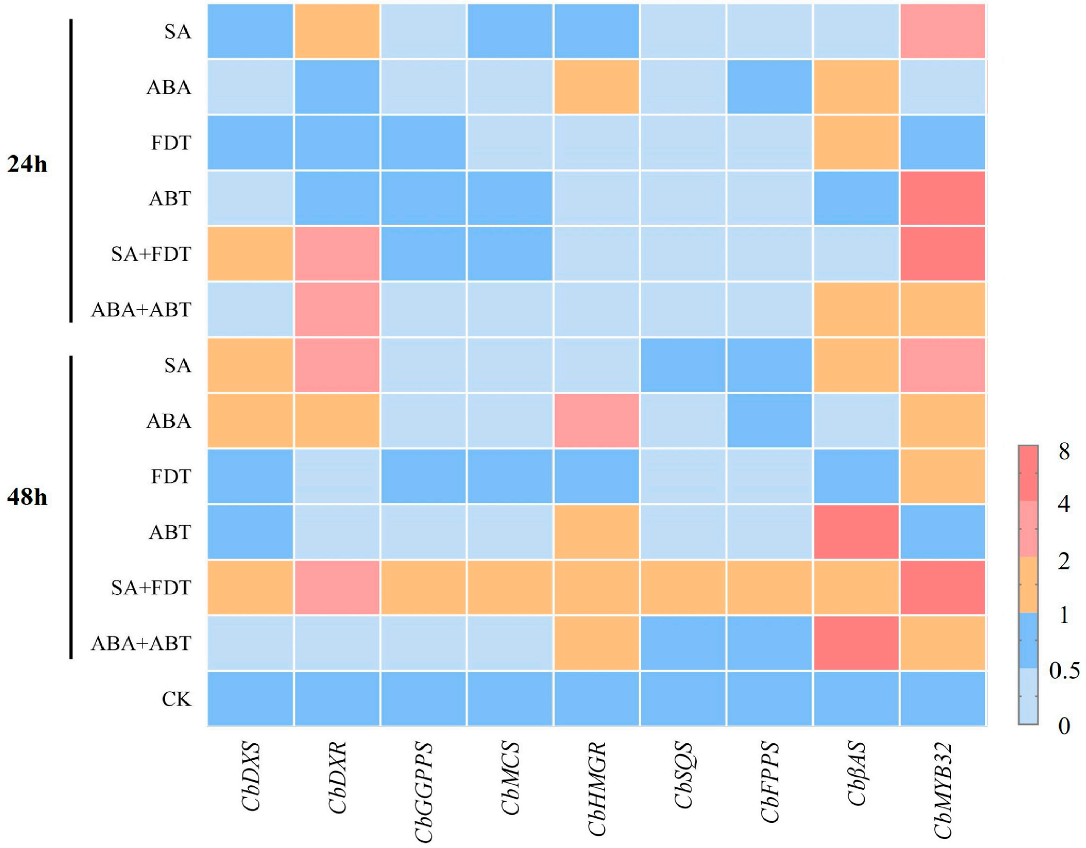

Supplemental Figure S3. Heat map of key enzymes gene expression in MEP pathways and *CbMYB32* after exogenous hormones treatment. The color gradient, ranging from blue, through yellow, to red represents values of genes expression. Fluridone (FDT) is an inhibitor of ABA, 1-Aminobenzotriazole (ABT) is an inhibitor of SA.

Supplemental Table S1. Prime sequence.

Name	Sequence (F:5'-3' , R:3'-5')
CbMYB32-pCambia1300	F: gagaacacgggggacgagctcATGTAGTATTGCGGATTTAGGGTTG<br>R: gcccttgctcaccatggtaccTTATAGTTGTTCTGAATTTATAAATTCCCA
CbMYB32-pGBKT7	F: ccgggaattcggcctccatggATGTAGTATTGCGGATTTAGGGTTG<br>R: tcagaggaggacctgcatatgTTATAGTTGTTCTGAATTTATAAATTCCCA
CbMYB32-$pTRV_2$	F: ggatcttccagagatATGTAGTATTGCGGATTTAGGGTTG<br>R: ctgccgttcgacgatCAAAGTCTCCTCTCTTTATATTTGGACG
CbPDS-$pTRV_2$	F: ggatcttccagagatGGGGTTTGTTAAAGATTGAAGAGC<br>R: ctgccgttcgacgatCCCTCGACGGCTTCCTCC
CbDXS-$pTRV_2$	F: ggatcttccagagatTCTTTCCTACAAAGAGGTTATGATCAA<br>R: ctgccgttcgacgatATGTGCATGAGCTCGGACTCA

Supplemental Table S2. Gene ID and GenBank of the reported TF sequences regulating terpenoids.

Gene ID	GenBank	Gene ID	GenBank	Gene ID	GenBank
SmMYB9	AGN52143.1	*CrWRKY1*	ADT82685.1	*BpbHLH9*	A0A1X9RU20
SmMYB36	AGN52170.1	*AaORA*	AGB07586.1	*AaNAC1*	AQU15092.1
SmMYB1	AGN52135.1	*AaERF1*	AEQ93554.1	*CsERF1*	AAV66332.1
BpMYB61	KT344120	*AaERF2*	AEQ93555.1	*SlNAC4*	AGH20612.1
BpMYB21	XP_018851905.1	*AaHY5*	JAT51023.1	*AaHDR*	ARA24921.1
AaEIN3	PWA84782.1	*AabHLH1*	A0A3S9XA60	*AcNAC3*	KF319052.1
